# Gut *Bifidobacteria* enrichment following oral *Lactobacillus*-supplementation is associated with clinical improvements in children with cystic fibrosis

**DOI:** 10.1186/s12890-022-02078-9

**Published:** 2022-07-28

**Authors:** Kathryn J. Ray, Clark Santee, Kathryn McCauley, Ariane R. Panzer, Susan V. Lynch

**Affiliations:** 1 Department of Epidemiology and Biostatistics, University of California San Francisco (UCSF), San Francisco, USA; 2 Francis I Proctor Foundation, University of California San Francisco (UCSF), San Francisco, USA; 3 Division of Gastroenterology, Department of Medicine, University of California San Francisco (UCSF), 513 Parnassus Ave, S357D, San Francisco, CA 94143 USA

**Keywords:** Cystic fibrosis, microbiome, *Lactobacillus rhamnosus* GG

## Abstract

**Background:**

Relationships between gut microbiomes and airway immunity have been established in murine and human studies of allergy and asthma. Early life *Lactobacillus* supplementation alters the composition and metabolic productivity of the gut microbiome. However, little is known of how *Lactobacillus* supplementation impacts the gut microbiota in children with cystic fibrosis (CF) and whether specific microbiota states that arise following gut microbiome manipulation relate to pulmonary outcomes.

**Methods:**

Stool samples were collected from CF patients enrolled in a multi-center, double-blind, randomized placebo-controlled trial of daily *Lactobacillus rhamnosus* strain GG (LGG) probiotic supplementation over a 12-month period. Fecal 16S rRNA biomarker sequencing was used to profile fecal bacterial microbiota and analyses were performed in QiiME.

**Results:**

*Bifidobacteria*-dominated fecal microbiota were more likely to arise in LGG-treated children with CF (*P* = 0.04). Children with *Bifidobacteria*-dominated gut microbiota had a reduced rate of pulmonary exacerbations (*IRR* = 0.55; 95% CI 0.25 to 0.82; *P* = 0.01), improved pulmonary function (+ 20.00% of predicted value FEV^1^; 95% CI 8.05 to 31.92; *P* = 0.001), lower intestinal inflammation (Calprotectin; *Coef* =  − 16.53 μg g^−1^ feces; 95% CI − 26.80 to − 6.26; *P* = 0.002) and required fewer antibiotics (IRR = 0.43; 95% CI 0.22 to 0.69; *P* = 0.04) compared to children with *Bacteroides-*dominated microbiota who were less likely to have received LGG.

**Conclusions:**

The majority of pediatric CF patients in this study possessed a *Bacteroides*- or *Bifidobacteria*-dominated gut microbiota. *Bifidobacteria*-dominated gut microbiota were more likely to be associated with LGG-supplementation and with better clinical outcomes.

**Supplementary Information:**

The online version contains supplementary material available at 10.1186/s12890-022-02078-9.

## Background

Mutations in the cystic fibrosis transmembrane conductance regulator, the genetic hallmark of cystic fibrosis (CF), leads to increased mucus secretion across organ systems and mucosal surfaces resulting in altered microbial colonization. CF patients experience chronic pulmonary inflammation punctuated by pulmonary exacerbations primarily due to acute microbial infection, resulting in reduction in pulmonary function with disease progression. Because pulmonary exacerbations represent a major clinical manifestation of the disease, much effort has appropriately been focused on this organ system. However, mucin secretion abnormalities associated with CF are system-wide, including in the gastrointestinal tract [1] and CF patients commonly experience failure to thrive and low BMI, phenotypes associated with gut microbiome dysfunction [2, 3]. In addition, in an attempt to manage acute pulmonary infections, this patient population is frequently administered antimicrobials, resulting in gut microbiome perturbations which have been described both in adult [4, 5] and pediatric [6] patients with CF.

The gut microbiome has emerged as a key player in regulating host immunity [4], protecting against pathogen overgrowth [5], providing energy to the human host [7] and reacting to or modifying specific drugs, including antimicrobials [8]. More recently its role in modulating airway mucosal and hematopoietic immunity has been revealed in murine studies [9, 10]. In human cohorts relationships between early life gut microbiota and subsequent airway disease development have been observed and more recent studies have indicated that at least in early life, *Lactobacillus rhamnosus* GG supplementation modifies the microbiota composition of high-risk for asthma infants [7]. Thus, the emerging view is that the gut microbiome may represent a modifiable mediator of immune function that influences susceptibility to airway infection.

Recent studies in adults with CF demonstrated dysbiosis in the gut microbiota as well as measurable changes in functionality of the gut microbiota compared to non-CF controls [4, 5]. However, an association between the gut-microbiota and lung function or other clinical outcomes in CF patients has not been shown. Conflicting results from studies indicate that *Lactobacillus rhamnosus* GG (LGG) supplementation is effective in reducing pulmonary exacerbations in CF patients [8, 11–15]. At the cellular level it’s been shown that LGG supplementation affects the expression of genes related to immune response and inflammation in humans [16]. Within Cystic Fibrosis patients, it’s been reported that probiotic supplementation improves intestinal inflammation and function [11, 12, 17], and reduces the rate of pulmonary exacerbations [13–15], but there has been inconsistent findings for other clinical outcomes such as pulmonary function, hospitalizations, or days of prescribed antibiotics [13, 15, 18]. A recent randomized clinical trial found a lack of evidence that *Lactobacillus rhamnosus* strain GG (LGG) probiotic supplementation was effective in reducing pulmonary exacerbations and hospital admissions in children with Cystic Fibrosis overall [8]. LGG may be associated with changes in the gut microbiota in some but not all children, for several reasons including the composition of the pre-treatment gut microbiome [19, 20], lack of adherence to treatment, diet, or other environmental factors.

Here, we hypothesized that the composition of the pediatric CF gut microbiota is altered by LGG supplementation in some but not all subjects and that distinct post-supplementation microbiota would be evident and related to clinical features of disease. Using samples collected from a multi-center, double-blinded, randomized placebo-controlled trial (which showed no statistically significant association between treatment and clinical outcomes [8]), we assessed whether, rather than LGG supplementation per se, the specific type of microbiome that arises following daily probiotic supplementation relates to the primary and secondary clinical endpoints in this population.

## Methods

### Study design

The methods for the randomized clinical trial have been discussed in detail previously [8]. The University Federico II, Naples, Italy, ethical committee approved the study in accordance with the Declaration of Helsinki. Briefly, children aged 2 to 16 years with a confirmed diagnosis of CF were enrolled and randomized to receive daily oral supplementation of either LGG or placebo, and were followed for 12 months. Compliance was evaluated with the parents/caregivers of the enrolled children by counting empty and full capsules, however we do not have access to the compliance data.

Clinical outcomes and stool samples were obtained at the time of randomization as well 12 months after treatment was started. Clinical outcomes assessed included number of pulmonary exacerbations, pulmonary function measured by the patients first forced expiratory volume (FEV^1^), intestinal inflammation (fecal calprotectin), and hospitalizations within the preceding 6-months (yes/no).

### Sample collection and processing

Stool swabs were transported in a nucleic acid preservative (RNALater, Ambion, CA) on dry ice. DNA was extracted from stool samples suing the MoBio fecal extraction kit (MoBIO, CA). The 16S rRNA gene was amplified (using extracted DNA as template) with universal bacterial primers Bact-27F 5′-AGAGTTTGATCCTGGCTCAG-3′ and Bact-1492R 5′-GGTTACCTTGTTACGAC TT-3′ and the high fidelity Takara Taq polymerase (Takara Mirus Bio Inc., WI). Reaction mixtures (50 μl final volume) contained 5 μl 10 × PCR buffer, 5 μl dNTPs (10 mM), 5 μl forward primer and reverse primer (25 pmol), 1 μl l Takara Taq polymerase (5 U μl^−1^) and 100 ng of template DNA. PCR was performed using an Eppendorf MasterCycler gradient PCR machine (Eppendorf, NY). Amplified products from samples were pooled using using the QIAquick gel extraction kit (Qiagen, CA) and DNA concentration determined by gel electrophoresis using the Invitrogen Low Mass Ladder (Invitrogen, CA). Following quantification and standardization of sample concentrations, each sample will be spiked with known concentrations of control oligonucleotides (ranging from 5.02 × 108 and 7.29 × 10^10^ molecules) that act as internal standards for normalization.

### Statistical analysis

Continuous baseline characteristics were compared between the two treatment groups using Wilcoxon rank-sum and Fishers exact tests for categorical methods in StataIC (ver. 13). Richness (number of OTU’s), evenness (Pielou’s), and Phylogenetic (Faith’s) diversity indices were chosen to measure alpha diversity (measure to estimate diversity within a sample) by arm and visit. Beta Diversity (measure to estimate diversity between samples) was measured using weighted unifrac, unweighted unifrac, Bray Curtis, and Canberra indices. All diversity indices were constructed as distance matrices using QIIME (14). Relationships between bacterial community composition (beta diversity matrices) and clinical outcomes, dominant genus, family, order, and treatment were assessed using *adonis* found in the R-package, vegan. To assess if dominant genus was associated with the clinical outcomes reported at baseline and 12-month visits, we used a robust mixed effect model (negative binomial for count data, linear for continuous data, and logistic for binary data), clustering by patient, and controlling for age, gender, and sputum organisms. A zero-inflated negative binomial model was chosen for modeling the counts of exacerbations due to over dispersion and excess of zeros, while negative binomial was chosen for days of prescribed antibiotics. And a Fishers Exact test was used to compare dominant taxa found in samples treated with placebo versus probiotics.

## Results

### Study cohort characteristics

**Fig. 1:**
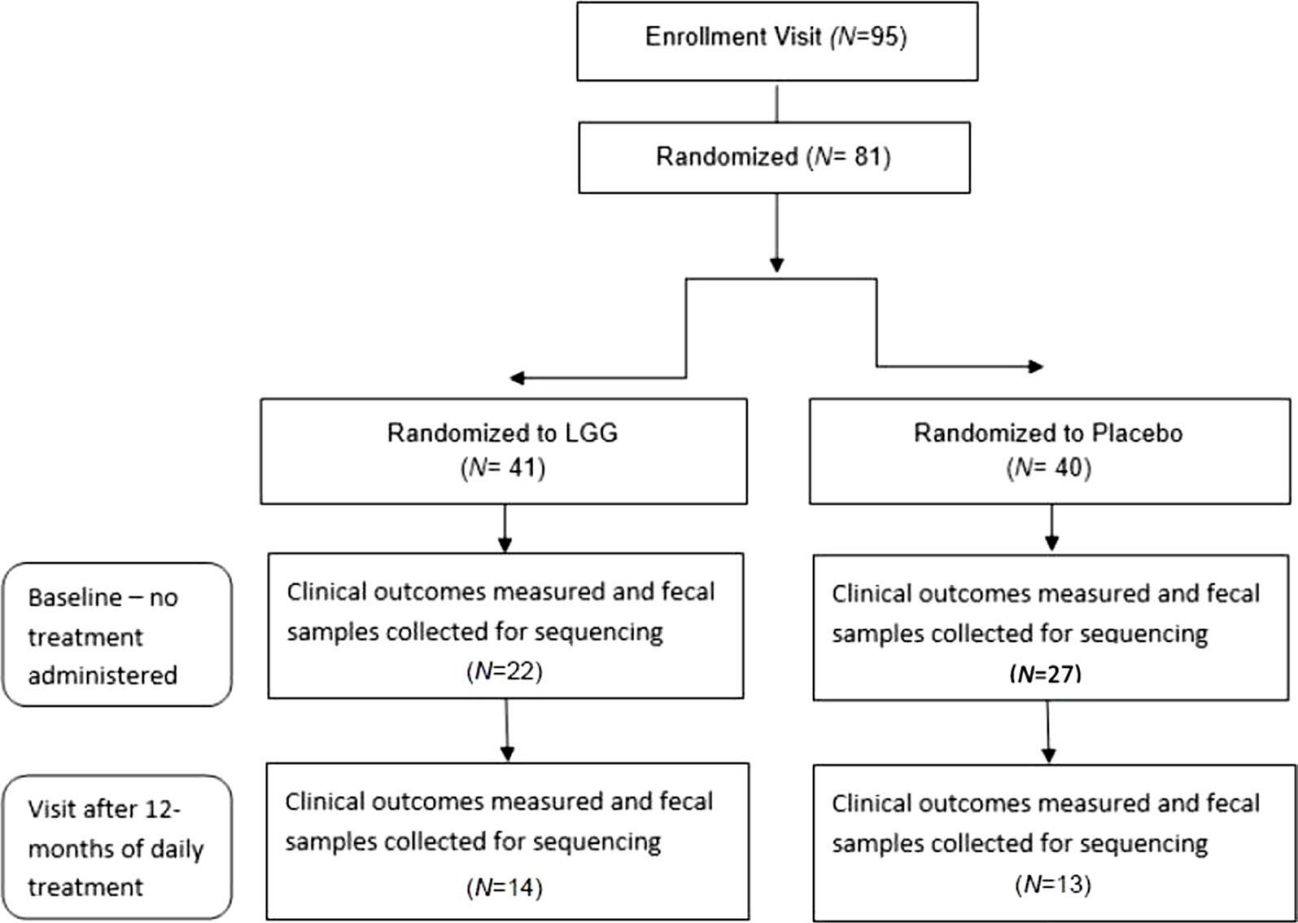
Flowchart

At the baseline (pre-treatment) visit, 50 patients had fecal samples collected and profiled for bacterial microbiota. Of these, 44% were randomized to LGG treatment (*N* = 22) and 54% to placebo (*N* = 27; Fig. 1) and 1 was missing treatment assignment data; enrollment sites included Firenze (*N* = 17, 34%), Milano (*N* = 19, 38%), and Napoli (*N* = 14, 28%), Italy. Baseline demographic, clinical and microbiota characteristics are outlined in Table 1; no significant difference was identified at baseline between those who were randomized to the LGG or placebo arms of the trial. Amongst those enrolled, 25 were male, 24 were female, with an overall mean age of 8.4 years and a mean BMI of 17.3 kg/m^2^. Clinical culture identified *Staphylococcus aureus*, *Pseudomonas aeruginosa* and *Stenotrophomonas maltophilia* as the predominant bacterial pathogens cultured from sputum, however at baseline, no significant difference in the frequency of culture-positivity for these pathogens was observed between treatment groups.

**Table 1:** Baseline characteristics measured prior to treatment assignment of patients who had fecal samples processed

Baseline characteristic	LGG (*N* = 22)	Placebo (*N* = 28)	Total^c^	*p*^a,b^
*Demographics*				
Sex, No. males (%)	10 (45%)	16 (57%)	26 (52%)	0.57
Age (years), median (IQR)	9.5 (4.2, 13.0)	7.1 (4.0, 12.3)	7.4 (4.1, 12.8)	0.39
Weight (kg), median (IQR)	32 (18.8, 40.2)	24.3 (16.3, 40.7)	30.7 (17.5, 40.2)	0.39
Height (cm), median (IQR)	140.0 (110.0, 153.0)	123.5 (101.5, 152.0)	128.0 (105.0, 153.0)	0.23
BMI Z-scores^b^, mean (SD)	0.06 (1.15)	0.60 (1.07)	0.36 (1.13)	0.10
*Clinical features*				
Pulmonary exacerbations, yes/no (%)	12/10 (55%)	22/6 (79%)	34/16 (68%)	0.13
FEV1 (median, IQR)	88.8 (67.5, 96.1)	89.6 (80.0, 108)	89.6 (80, 100)	0.42
Calprotectin (median, IQR)	77.0 (35.0, 240.0)	71.9(15.0, 138.0)	75.0 (28.0, 149.7)	0.37
Days on antibiotics in past 6 mos, median (IQR)	7 (0, 22)	15 (2, 25)	10 (0, 24.5)	0.18
Hospitalizations in past 6 mos, yes/no (%)	3/19 (14%)	3/25 (11%)	6/44 (12%)	1
*Microbiological*				
Sputum organism				
*P. aeruginosa,* yes/no (%)	5/16 (24%)	2/24 (8%)	7/40 (15%)	0.22
*S. aureus*, yes/no (%)	5/15 (25%)	11/14 (44%)	16/29 (36%)	0.22
*S. maltophilia*, yes/no (%)	1/20 (5%)	5/21 (19%)	6/41 (13%)	0.20

^a^Continuous and categorical characteristics compared using Wilcoxon rank-sum and Fishers exact tests, respectively

^b^BMI Z-scores compared using T-test

^c^50 Baseline samples were processed, but treatment assignment was missing for 1 sample at baseline

**Fig. 2:**
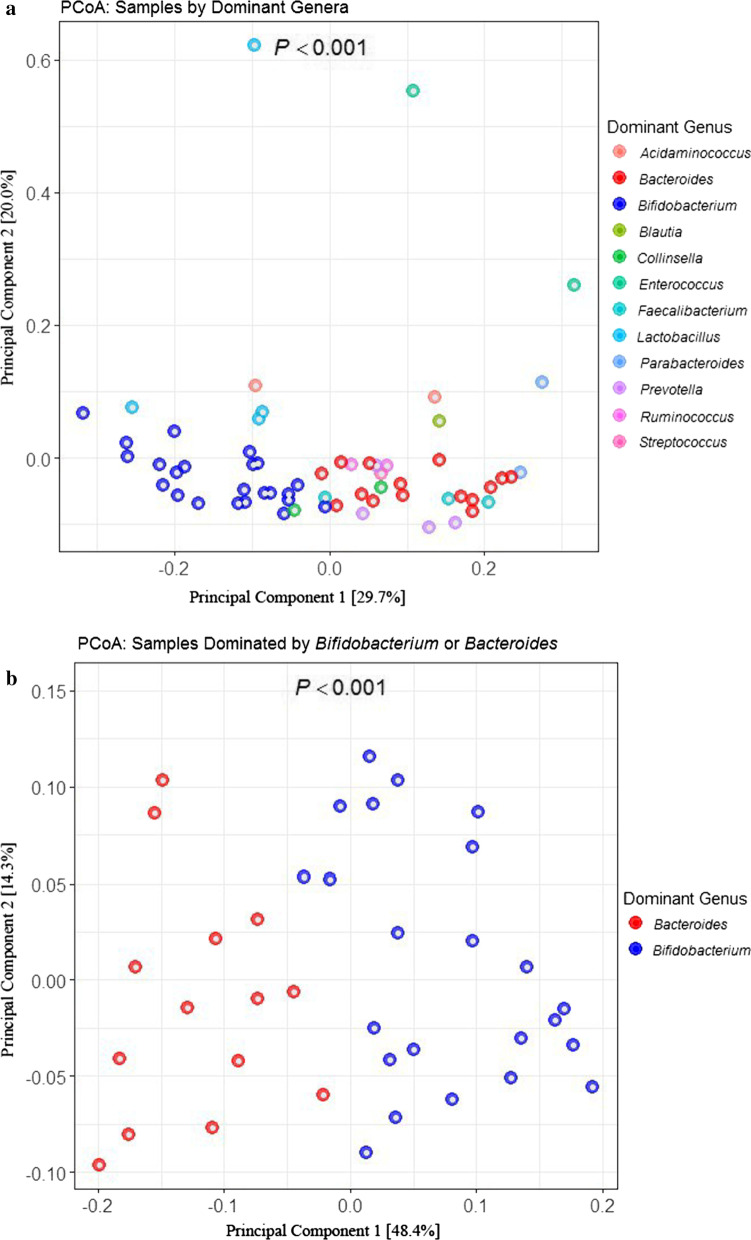
**a** PCoA showing weighted UniFrac beta diversity for all baseline and 12-month samples color-coded by dominant genus found in the sample. Principal components 1 and 2 [amount of variance explained by PC] are shown on the X and Y axis respectively. **b** PCoA showing weighted UniFrac beta diversity for all baseline and 12-month samples that were either dominated by Bacteroides or Bifidobacterium

Upon examination of the gut microbiota of baseline samples (n = 49) we found no significant association between microbiota composition and treatment arm or any measured clinical factors, but did note that the dominant bacterial genus present explained a large proportion of variance in community composition (Permanova, R^2^ = 0.62, *P* = 0.001; Table 2, Fig. 2). Further examination of this data indicated that the majority of patients were dominated by taxa belonging to the *Bifidobacteria* (32%, N = 16) or *Bacteroides* (18%, N = 9), with smaller proportions of subjects exhibiting *Prevotella* (8%, N = 4), *Lactobacillus* (6%, N = 3), *Enterococcus* (4%, N = 2), *Acidaminococcus* (2%, N = 1), *Blautia* (2%, N = 1), *Collinsella* (2%, N = 1), *Coprococcus (*2%, N = 1), *Faecalibacterium* (2%, N = 1), *Parabacteroides* (2%, N = 1), *Proteus* (2%, N = 1), *Ruminococcus* (2%, N = 1) and Unidentified Genera (16%, N = 8).

**Table 2:** Permutation Analysis of Variance (PERMANOVA) results using Adonis in the R environment to determine factors that significantly (*P* < 0.05) explained variation in microbiota beta diversity

	*N*	R^2^	*P* value
*Baseline*			
Dominant genus* (category)	50	0.62	0.0001
Exacerbations, *(N*)	50	0.01	0.81
Hospitalization (yes/no)	50	0.01	0.70
Calprotectin (μg/g of feces)	46	0.01	0.91
Days prescribed antibiotic coverage (*N*)	50	0.01	0.73
Treatment (LGG or Placebo)	49	0.03	0.64
*12-Month visit*			
Dominant genus* (category)	27	0.53	0.0001
Exacerbations, *(N*)	19	0.18	0.003
Hospitalization (yes/no)	19	0.13	0.02
Calprotectin (μg/g of feces)	23	0.09	0.048
Days prescribed antibiotic coverage (*N*)	19	0.19	0.001
Treatment (LGG or Placebo)	26	0.03	0.71

*Based on a weighted Unifrac distance

### LGG supplementation of CF patients does not lead to significant differences in fecal alpha diversity

Of the 95 CF patients enrolled in the trial, samples for stool microbiota analyses included 50 at baseline and 27 at 12-months of which 24 represented paired repeated samples from individual patients. Data describing treatment assignment for 1 patient of the 24 paired repeated samples was also missing. Among the 23 patients with complete data 11 were treated for with LGG probiotics for 1 year and 12 were treated with placebo for 1 year. Alpha diversity indices, including richness (number of observed operational taxonomic units; OTUs), Pielou’s evenness (distribution of OTUs within a sample) and Faith’s phylogenetic diversity (which incorporates community richness, evenness and phylogenetic relatedness) were calculated for each sample and compared between the treatment groups cross-sectionally using all samples available and longitudinally, using the sub-set of paired samples (Additional file 1: Table S2). No significant differences in fecal alpha diversity was observed between treatment groups when all samples were considered, or when paired samples were examined by linear mixed effect model testing (Additional file 1: Table S2). However, we noted that, even in this small study, LGG-treated children exhibited a trend towards increased fecal microbiota alpha diversity over time (Additional file 2: Fig. S1).

### CF fecal beta diversity is related to dominant bacterial genus and 12-month clinical outcomes

Because the effect of LGG supplementation has recently been shown to impact only a discrete population of bacterial taxa within the early-life gut microbiota [7], we hypothesized that this may also be true in pediatric CF patient populations. To address this hypothesis, we first examined fecal bacterial beta-diversity to determine whether significant compositional differences in gut microbiota existed within the baseline-visit samples or 12-month-visit samples. The largest proportion of variance (R-squared) was attributed to the dominant bacterial genus in each samples at both baseline or 12 month visits (Table 2). This indicates that each dominant bacterial genus co-associates with a distinct group of bacteria, and that these relatively reproducible gut microbiota structures explain a large proportion of variance in gut microbiota profiles of pediatric CF patients in our study (Fig. 2). Next, we examined the relationship between beta diversity and clinical outcomes at each visit. There were no associations between baseline gut microbiota beta diversity and clinical outcomes also measured at baseline. However, at 12 months, by the end of the supplementation period, a number of clinical outcomes measured at the 12-month visit significantly associated with gut bacterial beta-diversity at this timepoint. These included the number of exacerbations (R^2^ = 0.18, *P* = 0.003), hospitalization (R^2^ = 0.13; *P* = 0.02), fecal calprotectin concentration (R^2^ = 0.09; *P* = 0.048), and days of prescribed antibiotic coverage (R^2^ = 0.19; *P* = 0.001; Table 2). These data were largely consistent irrespective of whether a weighted UniFrac, Canberra, or Bray–Curtis distance matrix was employed (Additional file 3: Table S1).

## Gut microbiota dominated by *Bifidobacterium* or *Bacteroides* differentially associate with clinical outcomes

Since the bacterial genus dominating the microbiota in our pediatric CF patient explained the largest proportion of beta-diversity variance and microbiota composition was related to clinical outcomes at the end of the intervention period, we next determined which bacterial genera dominated pediatric CF patient gut microbiota. Amongst all baseline and 12-month fecal samples profiled (*N* = 77), the majority were dominated by *Bifidobacterium* (29%; *N* = 22) or *Bacteroides* (19%, *N* = 15; Fig. 2). The rate of pulmonary exacerbations among those patients with fecal microbiota dominated by *Bifidobacterium* was 0.54 less than those dominated with *Bacteroides* (IRR = 0.45; 95% CI 0.25 to 0.82*; P* = 0.01; Table 3) shown in Table 3. Consistent with this observation, patients with gut microbiota dominated by *Bifidobacterium* had 20.0% increase in FEV^1^ compared to those dominated by *Bacteroide*s (Coef = 20.00; 95% CI 8.05 to 31.92; *P* = 0.001; Table 3). Patients with *Bifidobacterium-*dominated gut microbiota also had significantly lower concentrations of fecal calprotectin (− 16.53 μg g^−1^ feces; − 26.80 to − 6.26; *P* = 0.002), and fewer days of prescribed antibiotics (IRR = 0.47; 95% CI 0.22 to 0.97; *P* = 0.04; Table 3).

**Table 3:** Comparison of clinical outcomes in those patients who had gut dominated by bifidobacterium versus bacteroides genera using mixed effect models correcting for age, gender, sputum organisms (except for FEV^1^) and clustering by patient

Clinical outcomes	Dominant genus				*Bifidobacterium* versus *Bacteroides* (95% CI)	*P*
	*Bifidobacterium*		*Bacteroides*			
	*N*	Adjusted estimate ± sd	*N*	Adjusted estimate ± sd		
Pulmonary exacerbations (*N*)	19	1.6 ± 1.5	13	2.9 ± 1.6	IRR = 0.55 (0.25 to 0.82)	0.01
FEV_1_ (percentage of predicted value)	22	103.48 ± 1.39	15	83.97 ± 1.48	Coef = 20.00 (8.05 to 31.92)	0.001
Calprotectin (µg/g of feces)	19	80.05 ± 61.43	13	107.73 ± 37.97	Coef = − 16.53 (− 26.80 to − 6.26)	0.002
Days of prescribed antibiotics in past 6-months (*N*)	19	25.8 ± 6.8	13	60.3 ± 5.9	IRR = 0.43 (0.22 to 0.69)	0.04

Data includes dominant genus and clinical outcomes reported at baseline and 12-month visits

### Distribution of dominant genus differs by LGG treatment

In the 23 paired samples sequenced at baseline and 12-months, there was a statistically significant difference between treatment arms when comparing the distribution of dominant genus found in the microbiome after 12 months of treatment (*P* = 0.005). No difference in the distribution of dominant genus was evident at baseline prior to treatment (*P* = 1). Patients with undefined genus at either visit (n = 7) were excluded from the analysis. All taxonomic ranks were tested to account for any questions about undefined taxonomic levels within samples. The demonstrated difference between treatment groups held for taxa levels: order (*P* = 0.02), class (*P* = 0.016), and phylum (*P* = 0.03), but not at the family level (*P* = 0.11). No difference in the distribution of dominant taxa was evident at baseline prior to treatment indicating the randomization balanced treatment between the smaller set of 23 subjects with paired data; family (*P* = 1), order (*P* = 1), class (*P* = 1), and phylum (*P* = 0.7).

Those treated with LGG transitioned to either *Bifidobacterium*- (*N* = 4), *Faecalibacerium*- (*N* = 2), *Collinsella-* (*N* = 1), or *Acidaminococcus*-dominated (N = 1) microbiota following 12-months of LGG supplementation (4 samples were undefined at the genus level). In comparison, the majority of placebo-treated patients transitioned to a *Bacteroides*- (*N* = 4), *Parabaceroides*- (*N* = *1*),* Streptococcus-* (*N* = 1)*, Enterococcus-* (*N* = 1)*, Ruminococcus-* (*N* = 1), *Proteus-* (*N* = 1), or *Bifidobacterium*-dominated (N = 1) gut microbiota within this same 12-month period (Additional file 4: Fig. S2). These data suggest that daily supplementation with LGG promotes development of distinct gut microbiota, more frequently dominated by *Bifidobacteria*.

## Discussion

Our analysis of the gut microbiota of 50 children aged 2–16 years with cystic fibrosis who underwent daily probiotic or placebo supplementation indicate there was not a statistically significant association in alpha or beta diversity across treatment groups. A relatively small number of distinct bacterial genera dominated the gut microbiota of the CF patients and explained the largest proportion of beta-diversity variance. Gut analysis based on these distinct microbiota states indicated that they differed in their exacerbation frequency, pulmonary function, intestinal inflammation and need for antibiotic treatment. LGG supplementation was associated with *Bifidobacterium*-dominated fecal microbiota, whereas placebo-supplemented patients typically exhibited *Bacteroides*-dominated microbiota. By the end of the intervention period, microbiota composition was related to clinical outcomes, with *Bifidobacteria*-dominated gut microbiota associated with decreased exacerbation, intestinal inflammation and antibiotic administration coupled with increased pulmonary function. Although, not statistically significant, LGG treatment increased alpha diversity over a period of 12 months. This is the first RCT that treated patients daily with LGG for an extended time period of 1 year. Sample size was limited at 12 months, which may have limited our ability to detect significant findings.

Beta-diversity variance related to several clinical outcomes as well as the dominant genus detected. Previous studies have shown that distinct bacterial microbiota compositions, each dominated by different bacteria, associate with clinical outcomes [21–23]. Here we strengthen that argument, showing that children with *Bifidobacteria*-dominated gut microbiota experience fewer exacerbations and hospitalizations, require less antibiotic coverage, and have decreased inflammation in the gut. Moreover, we show that LGG supplementation of pediatric CF patients is associated with increased prevalence of *Bifidobacteria-*dominated gut microbiota, offering a plausible explanation for why some children administered LGG in this trial exhibit improved clinical and immunological outcomes.

Limitations of this study include its small size and that it contains subgroup analyses which both limit the number of samples as well as generalizability of findings. Additionally, there were significantly less samples available from the 12-month visit, however balance of treatment groups was maintained in this smaller group. Additionally, extrinsic factors that can shape the gut microbiota such as diet were not captured in this study. Nonetheless, these findings suggest that daily supplementation with LGG is associated with *Bifidobacteria*-dominated fecal microbiota in some, but not all CF children and that *Bifidobacteria*-dominated microbiota are associated with improved clinical outcomes. These findings require validation in larger clinical trials. They may also offer an explanation for the varied response across CF patient populations to probiotic supplementation and provide early evidence that gut microbiome manipulation is associated with improvements in airway disease status in CF patients.

## Supplementary Information

**Additional file 1. Table S2.** Alpha diversity (Richness, Pielou's Evenness and Faith's phylogenetic diversity) indices of all samples analyzed cross-sectionally between treated and placebo groups.

**Additional file 2. Figure S1.** Alpha Diversity by treatment group over a 12 month period.

**Additional file 3. Table S1.** Beta Diversity: Permutation Analysis of Variance (PERMANOVA) results using Adonis in the R environment to determine factors that significantly (*P* < 0.05) explained variation in microbiota beta diversity.

**Additional file 4. Figure S2.** **a**–**e** Shift table showing composition of gut microbiota of patients (n = 23) having paired samples at baseline (displayed horizontally) and 12-month visit (displayed vertically) are shown at each taxonomic level. Red shading represents patients treated with LGG probiotics (*N* = 11), blue shading represents patient’s treated with placebo (*N* = 12). Patients with undefined taxonomy at either visit were excluded from the analysis. In the 23 patients (that had samples sequenced at baseline and 12-months), there was a statistically significant difference between the distribution of dominant taxa by treatment arms found in the microbiome after 12 months of treatment at the genera (*P* = 0.005), order (*P* = 0.02), class (*P* = 0.016), and phylum (*P* = 0.03) levels, but not at the family level (*P* = 0.11). All ranks were tested to account for any questions about undefined taxonomic levels within samples. No difference in the distribution of dominant genus was evident at baseline prior to treatment indicating the randomization held for this subset of patients that had fecal samples at 12-months at the genera (*P* = 1), family (*P* = 1), order (*P* = 1), class (*P* = 1), and phylum (*P* = 0.7)

## Data Availability

The datasets used and/or analysed during the current study are available from the corresponding author on reasonable request.
