# Supplementary material for: Gut Bifidobacteria enrichment following oral Lactobacillus-supplementation is associated with clinical improvements in children with cystic fibrosis
Source: BMC Pulm Med. 2022 Jul 28;22:287. doi: 10.1186/s12890-022-02078-9 (PMC9330662; doi:10.1186/s12890-022-02078-9)
Supplement: Supplementary file 1 — Additional file 1. Table S2. Alpha diversity (Richness, Pielou's Evenness and Faith's phylogenetic diversity) indices of all samples analyzed cross-sectionally between treated and placebo groups. [file 12890_2022_2078_MOESM1_ESM.docx]

| **Supplemental Table 2.** Alpha diversity indices of all samples cross-sectionally, representing Richness (observed OTU's), Evenness (Peilou's) and Phylogenetic (Faith's diversity of fecal microbiome samples between treated and placebo groups. | | | | | |
| --- | --- | --- | --- | --- | --- |
| **Diversity** | **Visit** | **Paired Data** | | ***P^1^*** | **Interaction *P*^2^** |
|  |  | **LGG (*N*=11)** | **Placebo (*N*=12)** |  |  |
|  |  | mean (min,max) | mean (min,max) |  |  |
| **Richness** | **Baseline** | 175 (12, 319) | 193(40, 268) | 0.73 | 0.24 |
|  | **12 months** | 204 (128,290) | 186 (133, 265) | 0.28 |  |
| **Evenness** | **Baseline** | 0.39 (0.07 to 0.48) | 0.41 (0.18 to 0.50) | 0.84 | 0.42 |
|  | **12 months** | 0.43 (0.36 to 0.49) | 0.42 (0.29 to 0.48) | 0.29 |  |
| **Phylogenetic Diversity** | **Baseline** | 17.4 (6.4 to 30.6) | 20.0 (9.6, 27.5) | 0.97 | 0.28 |
|  | **12 months** | 20.1 (14.5, 29.2) | 19.9(16.0, 28.2) | 0.26 |  |
| ^1^Paired T-test |  |  |  |  |  |
| ^2^Interaction term testing change in alpha diversity over time by treatment group | | | |  |  |
|  |  |  |  |  |  |
