# Supplementary figures and images for: Gut Bifidobacteria enrichment following oral Lactobacillus-supplementation is associated with clinical improvements in children with cystic fibrosis
Source: BMC Pulm Med. 2022 Jul 28;22:287. doi: 10.1186/s12890-022-02078-9 (PMC9330662; doi:10.1186/s12890-022-02078-9)

**Supplemental Figure 1.** Alpha Diversity by treatment group over a 12 month period.

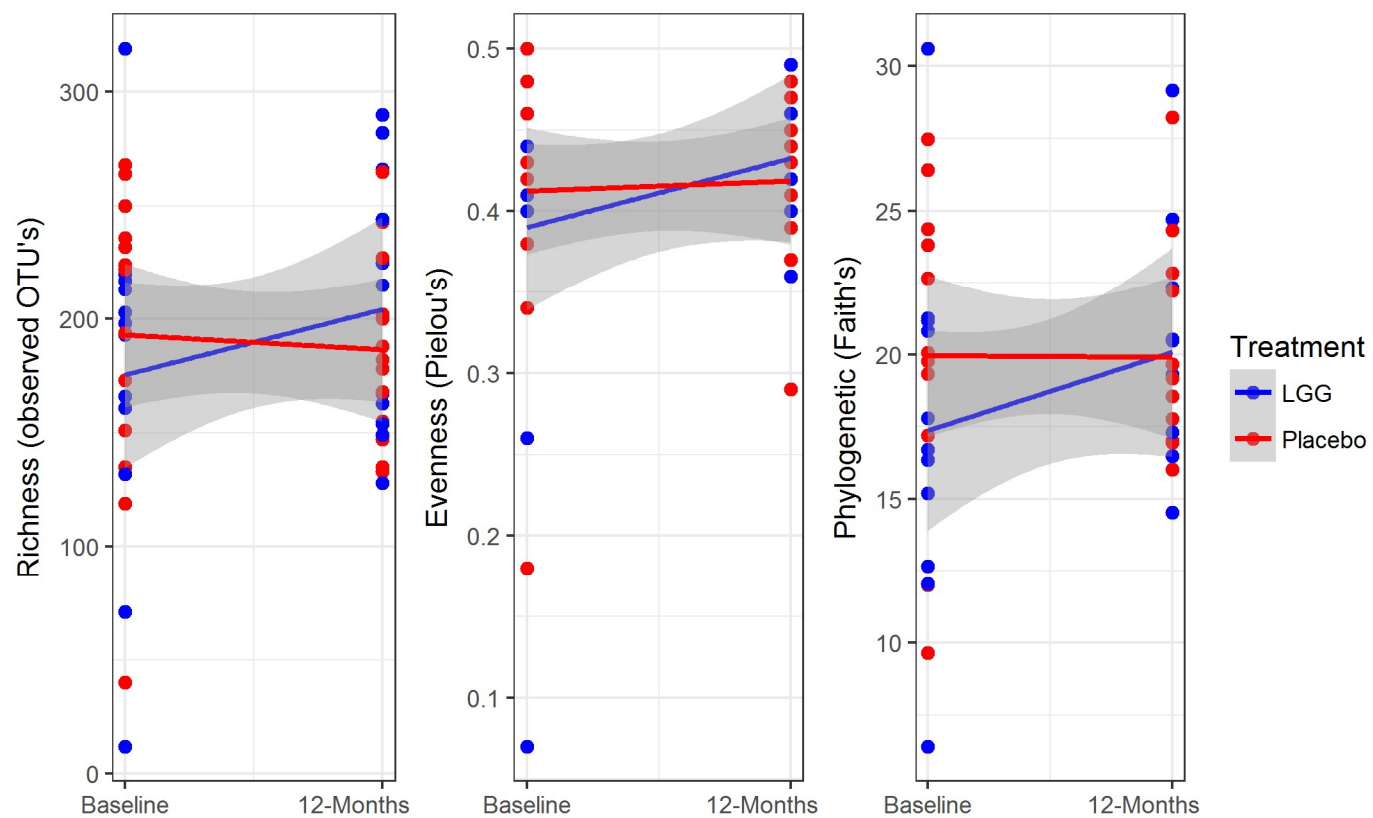

Supplement: Supplementary file 2 — Additional file 2. Figure S1. Alpha Diversity by treatment group over a 12 month period. [file 12890_2022_2078_MOESM2_ESM.pdf]
