# Supplementary material for: Gut Bifidobacteria enrichment following oral Lactobacillus-supplementation is associated with clinical improvements in children with cystic fibrosis
Source: BMC Pulm Med. 2022 Jul 28;22:287. doi: 10.1186/s12890-022-02078-9 (PMC9330662; doi:10.1186/s12890-022-02078-9)
Supplement: Supplementary file 3 — Additional file 3. Table S1. Beta Diversity: Permutation Analysis of Variance (PERMANOVA) results using Adonis in the R environment to determine factors that significantly (P < 0.05) explained variation in microbiota beta diversity. [file 12890_2022_2078_MOESM3_ESM.docx]

| **Supplemental Table 1. Beta Diversity:**  Permutation Analysis of Variance (PERMANOVA) results using Adonis in the R environment to determine factors that significantly (P<0.05) explained variation in microbiota beta diversity. | | | |
| --- | --- | --- | --- |
| **Unweighted Unifrac** | | | |
| **Baseline** | ***N*** | **R^2^** | ***P-*value** |
| Dominant Genus* (category) | 40 | 0.35 | 0.01 |
| Hospitalization (yes/no) | 50 | 0.04 | 0.04 |
| Treatment (LGG or Placebo) | 49 | 0.03 | 0.86 |
| **12-Month Visit** | ***N*** | **R^2^** | ***P-*value** |
| Hospitalization (yes/no) | 19 | 0.08 | 0.06 |
| Treatment (LGG or Placebo) | 26 | 0.03 | 0.71 |
| **Bray Curtis** | | | |
| **Baseline** | ***N*** | **R^2^** | ***P-*value** |
| Dominant Genus* (category) | 41 | 0.50 | 0.0001 |
| Hospitalization (yes/no) | 19 | 0.04 | 0.01 |
| Treatment (LGG or Placebo) | 49 | 0.04 | 0.55 |
| **12-Month Visit** | ***N*** | **R^2^** | ***P-*value** |
| Dominant Genus* (category) | 21 | 0.71 | 0.001 |
| Exacerbations, *(N*) | 19 | 0.13 | 0.047 |
| Hospitalization (yes/no) | 19 | 0.12 | 0.01 |
| Days Prescribed Antibiotic Coverage (*N*) | 19 | 0.10 | 0.004 |
| Treatment (LGG or Placebo) | 26 | 0.02 | 0.81 |
| **Canberra** | | | |
| **Baseline** | ***N*** | **R^2^** | ***P-*value** |
| Dominant Genus* (category) | 50 | 0.30 | 0.0001 |
| Hospitalization (yes/no) | 19 | 0.02 | 0.04 |
| Treatment (LGG or Placebo) | 49 | 0.04 | 0.63 |
| **12-Month Visit** | ***N*** | **R^2^** | ***P-*value** |
| Dominant Genus* (category) | 27 | 0.48 | 0.0001 |
| Exacerbations, *(N*) | 19 | 0.13 | 0.01 |
| Hospitalization (yes/no) | 19 | 0.11 | 0.01 |
| Days Prescribed Antibiotic Coverage (*N*) | 19 | 0.14 | 0.003 |
| Treatment (LGG or Placebo) | 26 | 0.02 | 0.81 |
