# Supplementary material for: Gut Bifidobacteria enrichment following oral Lactobacillus-supplementation is associated with clinical improvements in children with cystic fibrosis
Source: BMC Pulm Med. 2022 Jul 28;22:287. doi: 10.1186/s12890-022-02078-9 (PMC9330662; doi:10.1186/s12890-022-02078-9)
Supplement: Supplementary file 4 — Additional file 4. Figure S2. a–e Shift table showing composition of gut microbiota of patients (n = 23) having paired samples at baseline (displayed horizontally) and 12-month visit (displayed vertically) are shown at each taxonomic level. Red shading represents patients treated with LGG probiotics (N = 11), blue shading represents patient’s treated with placebo (N = 12). Patients with undefined taxonomy at either visit were excluded from the analysis. In the 23 patients (that had samples sequenced at baseline and 12-months), there was a statistically significant difference between the distribution of dominant taxa by treatment arms found in the microbiome after 12 months of treatment at the genera (P = 0.005), order (P = 0.02), class (P = 0.016), and phylum (P = 0.03) levels, but not at the family level (P = 0.11). All ranks were tested to account for any questions about undefined taxonomic levels within samples. No difference in the distribution of dominant genus was evident at baseline prior to treatment indicating the randomization held for this subset of patients that had fecal samples at 12-months at the genera (P = 1), family (P = 1), order (P = 1), class (P = 1), and phylum (P = 0.7) [file 12890_2022_2078_MOESM4_ESM.pdf]

2a. Taxonomic level = Genus

Key	
LGG	PLC

Taxonomy after 12-months of Treatment (rows) / Taxonomy at Baseline (Genus level) (columns)

Phylum	Class	Order	Family	Genus	*Bifidobacterium*	*Collinsella*	undefined	*Faecalibacterium*	undefined	*Acidaminococcus*	*Rumniococcus*	*Blautia*	undefined	undefined	undefined	*Streptococcus*	*Lactobacillus*	*Bacteroides*	*Parabacteroides*	*Prevotella*	*Proteus*	*Enterococcus*	undefined	LGG	PLC	*P*
Actinobacteria	*Actinobacteria*	*Bifidobacteriales*	*Bifidobacteriaceae*	***Bifidobacterium***	S103; S108					S148				S121				S133						4	1	
		Coriobacteriales	*Coriobacteriaceae*	***Collinsella***														S100						1	0	
				**undefined**																S151				0	1	
Firmicutes	*Clostridia*	*Clostridiales*	*Ruminococcaceae*	***Faecalibacterium***														S126				S119		2	0	
				**undefined**																				0	0	
			Veillonellaceae	***Acidaminococcus***					S129															1	0	
			Lachnospiraceae	***Rumniococcus***	S115																			0	1	
				Blauta																				0	0	
				**undefined**																	S122			1	0	
			Clostridiaceae	**undefined**																				0	0	0.005
			Undefined Family	**undefined**											S104									1	0	
	Bacilla	*Lactobacillales*	Streptococcaceae	***Streptococcus***																			S111	0	1	
			Lactobacillaceae	***Lactobacillus***																				0	0	
Bacteroidetes	*Bacteroidia*	*Bacteroidales*	*Bacteroidaceae*	***Bacteroides***	S149												S110	S117; S140						0	4	
			Porphyromonadaceae	***Parabacteroides***								S134												0	1	
			Prevotallaceae	***Prevotella***																				0	0	
Proteobacteria	*Gamma-proteobacteria*	*Enterobacteriales*	*Enterobacteriaceae*	***Proteus***	S112																			0	1	
				**Enterococcus**														S156						0	1	
				**Undefined**														S154	S152					1	1	
**Total number of subjects with longitudinal paired samples**																								**11**	**12**	

2b. Taxonomic level = Family

Key	
LGG	PLC

Taxonomy after 12-months of Treatment (rows) vs. Taxonomy at Baseline (Family level) (columns)

Phylum	Class	Order	Family	*Bifidobacteriaceae*	*Coriobacteriaceae*	*Ruminococcaceae*	*Veillonellaceae*	Undefined Family	*Clostridiaceae*	undefined	*Streptococcaceae*	*Lactobacillaceae*	*Bacteroidaceae*	*Porphyromonadaceae*	*Prevotallaceae*	*Enterobacteriaceae*	LGG	PLC	*P*
Actinobacteria	*Actinobacteria*	*Bifidobacteriales*	***Bifidobacteriaceae***	S103; S108			S148		S121				S133				4	1	0.11
		Coriobacteriales	***Coriobacteriaceae***										S100		S151		1	1	
Firmicutes	*Clostridia*	*Clostridiales*	***Ruminococcaceae***										S126			S119	2	0	
			Veillonellaceae			S129											1	0	
			Lachnospiraceae	S115												S122	1	1	
			Clostridiaceae														0	0	
			**Undefined Family**							S104							1	0	
	Bacilla	*Lactobacillales*	**Streptococcaceae**													S111	0	1	
			**Lactobacillaceae**														0	0	
Bacteroidetes	*Bacteroidia*	*Bacteroidales*	***Bacteroidaceae***	S149								S110	S117; S140				0	4	
			Prevotallaceae					S134									0	1	
Proteobacteria	*Gamma-proteobacteria*	*Enterobacteriales*	***Enterobacteriaceae***	S112									S156; S154	S152			1	3	
**Total number of subjecs with longitudinal paired samples**																	**11**	**12**	

2c. Taxonomic level = Order

Key	
LGG	PLC

				Taxonomy at Baseline (Order level)							
	Phylum	Class	Order	*Bifidobacteriales*	*Clostridiales*	*Lactobacillales*	*Bacteroidales*	*Enterobacteriales*	LGG	PLC	*P*
Taxonomy after 12-months of Treatment	*Actinobacteria*	*Actinobacteria*	***Bifidobacteriales***	S103; S108	S148 S121		S133		4	*1*	
			Coriobacteriales				S100 S151		1	1	
	Firmicutes	*Clostridia*	***Clostridiales***	S115	S129; S104		S126	S119; S122	5	1	0.02
		Bacilla	***Lactobacilliales***					S111	0	1	
	Bacteroidetes	*Bacteroidia*	***Bacteroidales***	S149	S134	S110	S117; S140		0	5	
	Proteobacteria	*Gamma-proteobacteria*	***Enterobacteriales***	S112			S156; S154 S152		1	3	
	**Total number of subjects with longitudinal paired samples**								**11**	**12**	

2 d. Taxonomic level = Class

Key	
LGG	PLC

			Taxonomy at Baseline (Class level)							
	Phylum	Class	*Actinobacteria*	*Clostridia*	*Bacilla*	*Bacteroidia*	*Gamma-proteobacteria*	LGG	PLC	*P*
Taxonomy after 12-months of Treatment	*Actinobacteria*	***Actinobacteria***	S103; S108	S148 S121		S133;S100	S151	5	2	
	Firmicutes	***Clostridia***	S115	S129; S104		S126	S119; S122	5	1	
		Bacilla					S111	0	1	0.016
	Bacteroidetes	***Bacteroidia***	S149	S134	S110	S117; S140		0	5	
	Proteobacteria	***Gamma-proteobacteria***	S112			S156; S154 S152		1	3	
	**Total number of subjecs with longitudinal paired samples**							**11**	**12**	

2e. Taxonomic level = Phylum

Key	
LGG	PLC

Taxonomy at Baseline (Phylum level)

Taxonomy after 12-months of Treatment	Phylum	*Actinobacteria*	*Firmicutes*		*Bacteroidetes*		*Proteobacteria*		LGG	PLC	*P*
	Actinobacteria	S103; S108	S148	S121	S133; S100			S151	5	2	0.03
	Firmicutes	S115	S129; S104		S126		S119; S122	S111	5	2	
	Bacteroidetes	S149	S134; S110		S117; S140				0	5	
	Proteobacteria	S112			S156; S154	S152			1	3	
	**Total number of subjecs with longitudinal paired samples**								**11**	**12**	

Supplemental Figure 2a-e. Shift table showing composition of gut microbiota of patients (n=23) having paired samples at baseline (displayed horizontally) and 12-month visit (displayed vertically) are shown at each taxonomic level. Red shading represents patients treated with LGG probiotics (*N*=11), blue shading represents patient's treated with placebo (*N*=12). Patients with undefined taxonomy at either visit were excluded from the analysis.

In the 23 patients (that had samples sequenced at baseline and 12-months), there was a statistically significant difference between the distribution of dominant taxa by treatment arms found in the microbiome after 12 months of treatment at the genera (*P*=0.005), order (P=0.02), class(*P*=0.016), and phylum (P=0.03) levels, but not at the family level (*P*=0.11) . All ranks were tested to account for any questions about undefined taxonomic levels within samples. No difference in the distribution of dominant genus was evident at baseline prior to treatment indicating the randomization held for this subset of patients that had fecal samples at 12-months at the genera (*P* =1), family (*P*=1), order (*P*=1), class (*P*=1), and phylum(*P*=0.7)
